# Purged from the Rolls: A Study of Medicaid Disenrollment in Iowa

**DOI:** 10.1089/heq.2019.0093

**Published:** 2019-12-16

**Authors:** Natoshia M. Askelson, Patrick Brady, Brad Wright, Suzanne Bentler, Elizabeth T. Momany, Peter Damiano

**Affiliations:** ^1^ Department of Community and Behavioral Health, University of Iowa College of Public Health, Iowa City, Iowa.; ^2^ Public Policy Center, University of Iowa, Iowa City, Iowa.; ^3^ Department of Family Medicine, University of North Carolina School of Medicine, Chapel Hill, North Carolina.

**Keywords:** Medicaid, evaluation, health insurance disenrollment

## Abstract

**Purpose:** To describe the impact of disenrollment from Medicaid because of failure to pay premiums as part of Iowa's Medicaid program's personal responsibility component.

**Methods:** We conducted a mixed method study consisting of in-depth interviews with disenrolled members in 2016 and 2017 (*n*=72) and a survey of disenrolled members in 2017 (*n*=225).

**Results:** Many disenrollees did not know why they were disenrolled, were unaware of the personal responsibility component or premium requirement, and were confused by the disenrollment process. Disenrollment had negative effects including stress, financial burden, and engaging in behaviors such as skipping medication and postponing medical or dental care. Furthermore, disenrollees were often unable to enroll in health insurance, and for those who did, many reported it was a difficult process.

**Conclusions:** Disenrollment had numerous, negative impacts on members who failed to pay their premiums. There was confusion about program requirements, which might indicate challenges communicating about a complicated program. Policymakers need to consider how to design and implement personal responsibility programs to achieve their desired outcome and reduce confusion and negative consequences.

## Introduction

Iowa is one of several states that designed their Medicaid expansion to include personal responsibility components using an 1115 Waiver.^1^ The Iowa Health and Wellness Plan (IHAWP) serves those who earn up to 138% of the federal poverty level (FPL) through health plans administered by managed care organizations. The personal responsibility component is the Healthy Behaviors Program (HBP), which requires individuals to complete a wellness examination and a health risk assessment annually. Failure to complete requirements triggers an income-based premium of $5 or $10 per month, and failure to pay premiums (or be granted a monthly exemption based on demonstrated financial need) results in disenrollment for individuals with incomes between 101% and 138% FPL.

A 2014–2015 analysis of HBP completion rates revealed low compliance with HBP requirements,^2^ suggesting that many individuals may face disenrollment in the next year. Reasons for the poor completion rate included limited awareness of program requirements among enrollees and providers and confusion owing to changes in program implementation.^3^ Moreover, simply increasing premiums can increase disenrollment from public health insurance programs.^4,5^ Disenrollment from public health insurance programs has negative consequences, including reduced access to health care,^6,7^ poorer health,^8^ increased financial burden,^6,7,9,10^ and limited ability to re-enroll in health insurance.^11^

To the extent that disenrollment creates hardships for individuals, preventing disenrollment would reduce those hardships and the administrative burden and costs associated with disenrolling and re-enrolling individuals. Because little is known about the disenrollment process and experience of being disenrolled and previous 1115 Waiver evaluations have not tackled these issues, it is important that we fill this gap in knowledge to better understand how to prevent disenrollment and mitigate the consequences. Within that context, we conducted a mixed methods study with individuals who had been disenrolled from IHAWP for failure to pay premiums. Our study aimed to answer three research questions: (1) What contributed to the individual being disenrolled? (2) How did they experience the disenrollment process? and (3) What were the consequences of disenrollment? Answering these questions can provide important insights into the design and implementation of Iowa's HBP and guidance for other states with—or contemplating—similar programs.

## Methods

Our mixed methods study is composed of in-depth interviews and a mailed survey.

### In-depth interviews of individuals disenrolled from IHAWP

To investigate individuals' experience of disenrollment and the extent to which it changed as the program matured, we conducted interviews with individuals disenrolled in 2016 and 2017. The Iowa Department of Human Services provided a monthly list of enrollees who were being disenrolled. In 2016, we randomly selected 200 of the 326 March disenrollees, and in 2017, we used all 184 February disenrollees. We sent the disenrollees a letter outlining the study, explaining the elements of informed consent, and inviting them to participate. We attempted to reach them by telephone (up to 10 attempts). After obtaining informed consent, interviewers asked participants open-ended questions about their disenrollment experience and the subsequent period. The interview guide was developed to address our research questions. Interviews were recorded and transcribed. Each participant was compensated. Review by an institutional review board is not required for 1115 Waiver evaluations as outlined by federal law.

We completed 37 interviews in 2016 and 35 interviews in 2017, for completion rates of 18.5% and 19.0%, respectively. We used a deductive approach to develop a codebook based on the interview guide, the research questions, and a preliminary reading of the transcripts.^12^ We had three coders in 2016 and four in 2017. We coded each transcript and identified relevant themes.^1^

### Survey of individuals disenrolled from IHAWP

We developed a survey based on the results of the interviews. Surveys were mailed on a rolling monthly basis from June to December 2017 to individuals who were disenrolled 3 months before for failure to pay premiums (e.g., surveys mailed in June were sent to members who were disenrolled on March 1). We excluded individuals who had participated in previous evaluation activities. Mailings varied in size as the monthly number of disenrolled individuals changed (Table 1).

**Table 1.** Individuals Eligible for and Completing the 2017 Iowa Health and Wellness Plan Disenrollment Survey by Month

Month survey was sent (month disenrolled)	Total	Completed	Response rate (%)
June (March)	130	36	28
July (April)	150	37	25
August (May)	2	1	50
September (June)	338	108	32
October (July)	229	55	24
Total	849	237	28
Adjusted^a^ total	749	237	32

^a^ Adjusted for ineligibles: removed respondents who no longer had a valid address or were out of Iowa.

Along with the survey, the packet included a cover letter that described the survey, indicated that participation was completely voluntary, and provided a telephone number to ask questions or opt out of the study. Respondents could complete the survey either on paper or online with a unique access code. To maximize response rates, each initial packet included a $2 bill, and respondents who returned a completed survey were sent a $20 gift card. After 1 week, we sent a postcard reminder, and after 4 weeks, we sent another survey packet. A total of 237 individuals returned completed surveys. After adjusting for ineligible respondents, the response rate was 32%. We had 12 households with two respondents each. We ran the analysis with one individual from each duplicated household randomly selected to be included. This resulted in an analytic sample size of *n*=225. We analyzed survey results by tabulating frequencies and generating descriptive statistics. Where appropriate, we conducted Pearson's chi-square tests. We then re-ran the analysis with the alternate individual from each household. The results were not different between the analyses, so we present the results from the first.

## Results

Because we found few differences between the 2016 and 2017 interview responses, we present the results aggregated across both time periods for the 72 people we interviewed. Table 2 presents demographic and other selected characteristics of the interview and survey respondents.

**Table 2.** Demographic Characteristics of 2016/2017 Disenrollment Interviews (*n*=72) and 2017 Iowa Health and Wellness Plan Disenrollment Survey Respondents (*n*=225)

Characteristics	Interview		Survey	
	*n*	%	*n*	%
Age category, years				
18–24	9	12.5	43	18.1
25–34	29	40.3	49	20.7
35–44	9	12.5	41	17.3
45–54	9	12.5	44	18.6
55–64	16	22.2	56	23.6
65 or older	0	0.0	3	1.3
Gender				
Male	29	40.3	89	39.7
Female	43	59.7	134	59.6
Race/ethnicity (check all that apply)				
American Indian/Alaskan Native	0	0.0	6	2.7
Asian	1	1.4	3	1.3
Hispanic/Latino	5	6.9	10	4.4
White	54	75.0	188	83.7
Black or African American	14	19.4	24	10.7
Education				
Less than high school	10	13.9	31	13.8
Graduated high school or equivalent	26	36.1	100	44.4
Greater than high school	36	50.0	94	41.8
Employment				
Employed	59	82.0	166	73.8
Unemployed	13	18.0	56	24.9
Experience with government programs (check all that apply)				
Supplemental Nutrition Assistance Program		N/A	107	47.6
Free or Reduced School Lunch Program		N/A	20	8.9
Supplemental Security Income		N/A	16	7.1
WIC^a^		N/A	4	1.8
Housing assistance		N/A	5	2.2
General assistance		N/A	8	3.6
Temporary assistance for needy families		N/A	27	12.0
Current health insurance status (check all that apply)				
Re-enrolled in IHAWP	29	40.3	31	13.8
Trying to re-enroll in IHAWP	7	9.7	24	10.7
Looking for health insurance	0	0.0	15	6.7
Purchased private health insurance	8	11.1	8	3.6
Waiting for employer health insurance	0	0.0	10	4.4
Have employer health insurance	4	5.6	21	9.3
On Medicaid/Title 19	0	0.0	14	6.2
On Medicare	0	0.0	8	3.6
Has no health insurance	31	43.1	102	45.3

^a^ Special Supplemental Nutrition Program for Women, Infants, and Children.

IHAWP, Iowa Health and Wellness Plan; N/A, not applicable.

### What led or contributed to the individual being disenrolled?

Most interviewees knew their disenrollment was owing to not paying premiums, but interviewees often did not understand the disenrollment process or what was required to prevent disenrollment. For example, some gave reasons besides failure to pay their premium for being disenrolled, including earning too much money or missing paperwork. Some believed they were wrongly disenrolled, saying they had completed the HBP requirements or claimed a financial hardship. Interviewees said that they did not receive bills or information in the mail. They said they would have liked more notice of disenrollment and believed they did not receive enough information or resources to avoid disenrollment.

When asked whether they were aware of the HBP before being disenrolled, only 24 interviewees said the program sounded familiar, and of those, only 12 felt confident in their response. The majority of interviewees (*n*=50) stated that they would have participated in the HBP had they been aware of it. Some interviewees could identify the source of their confusion about the program requirements: “Yeah, when I went to the doctors, I thought they automatically told them that I had did that part, but, you have to contact them. So that was probably bad communication on my part.” [793] and “I was doing basically that but I wasn't reporting it, because I thought the clinic would report it itself. And that's also what kinda hurt me.” [769].

Survey respondents gave various reasons for their disenrollment, but slightly under half correctly identified that they were disenrolled for not paying premiums and 1 in 10 did not know why they were disenrolled (Table 3). Roughly a quarter of the respondents had heard of the HBP and over half were unaware that they could claim a financial hardship (Table 4). Less than half of the respondents understood that they had to pay a monthly premium if they failed to meet HBP requirements (Table 3). Asked why they did not pay the premiums, ∼45% selected they did not have the money and nearly 20% selected forgot to pay (Table 3).

**Table 3.** Experience of Iowa Health and Wellness Plan Disenrollment Survey Respondents Through the Disenrollment Process

	*n*	%
How did you find out about being disenrolled? (*n*=194)		
Received a letter	147	75.8
Told when getting health care	17	8.8
Told when getting dental care	4	2.1
Told when getting a prescription	17	8.8
Other	7	3.6
Did you know you were going to be disenrolled before it happened? (*n*=225)		
Yes	46	20.4
No	145	64.4
Why did you think you were disenrolled? (check all that apply) (*n*=225)		
Did not pay premiums	109	48.4
Did not pay co-pays	16	7.1
Did not return proper paperwork	17	7.6
Made too much money	33	14.7
Don't know	27	12.0
Did you know that you owed a monthly premium? (*n*=225)		
Yes	103	45.8
No	116	51.6
Why did you not pay monthly premiums?(check all that apply; *n*=225)		
Did not know needed to pay	83	36.9
Did not have the money	100	44.4
Forgot to pay	39	17.3
Did not know how to pay or who to pay	13	5.8
Did not receive invoices or bills telling to pay	27	12.0
Did not understand invoices or bills telling to pay	19	8.4
While you had no health insurance coverage, did you?(check all that apply; *n*=225)		
Delay getting prescriptions filled	73	32.4
Stretch medicine so it would last longer	56	24.9
Stop taking prescribed medication	64	28.4
Not seek health care when needed	112	49.8
Delay seeking preventive medical care	87	38.7
Delay seeking dental care	81	36.0
Pay more for medical care, dental care, or prescriptions	46	20.4

**Table 4.** Comparison of Iowa Health and Wellness Plan Disenrollment Survey Respondents Awareness of the Financial Hardship Waiver, Awareness of the Healthy Behaviors Program Preparation for Disenrollment, and Action Taken After Being Disenrolled Between Those Who Did and Who Did Not Re-enroll in Any Health Insurance Program (*n*=225)

	Total		Re-enrolled		Not re-enrolled		Pearson's chi-square	*p*
	*n*	%	*n*	%	*n*	%		
Are you aware you could declare a financial hardship?								
Yes	88	39.1	36	40.9	52	59.9	2.75	0.26
No	135	60.0	44	32.6	91	67.4		
Are you aware of the Healthy Behaviors Program?								
Yes	59	26.2	24	40.7	35	59.3	1.00	0.61
No	166	71.6	54	33.5	107	66.5		
Did you do anything to prepare for being disenrolled?^a^								
Did something	24	10.7	13	54.2	11	45.8	4.06	0.04
Did nothing	201	89.3	67	33.3	134	66.7		
What did you do after you were disenrolled?^b^								
Did something	136	60.4	59	4334	77	56.6	9.19	<0.01
Did nothing	89	39.6	21	23.6	68	76.4		

^a^ Significant at α=0.05.

^b^ Significant at α=0.01.

### How did they experience the disenrollment process?

At the time of the interview, most interviewees knew they had been disenrolled, however 66.7% stated that being disenrolled surprised them. Three-quarters of interviewees found out about their disenrollment from a letter, but ∼1 in 10 interviews said they initially found out while trying to access services or fill prescriptions. One recalled, “I actually got an abscess in a tooth…And my dentist actually informed me with my upcoming appointment that I was disenrolled before I even got the letter. Somehow they knew. I don't know if they check before your appointment or what it is. But they told me that they could not see me.” [552].

For the survey respondents who answered the question about awareness of their disenrollment (*n*=217), 83.4% indicated that they were aware of being disenrolled at the time of the survey. Of the 194 respondents who indicated how they found out they had been disenrolled, three-quarters of the respondents reported learning about it from a letter, but only one-fifth knew they were going to be disenrolled before receiving notification (Table 3).

### What were the consequences of disenrollment?

#### On their health and wellbeing

Being disenrolled negatively impacted interviewees' ability to adhere to prescription medications. They reported not being able to refill prescriptions, not being able to see a doctor for new medications, and taking fewer doses of medication to make it last longer. Although for some the disenrollment period was relatively short, some interviewees reported needing medication daily and that going even a few weeks without health insurance could have a profound impact on their ability to manage their health. One interviewee provided an example: “I have emphysema. And my Advair, I called just to find out how much my Advair was. It's 500 dollars a month. I only make 1,200 dollars a month. So that's almost half my monthly income would go just for my medicine.” [723].

Dental issues were the third most-cited health concern among interviewees, behind weight and high blood pressure. A lack of coverage directly resulted in dental needs being unmet, as described by one interviewee: “…I have bad teeth and I need my wisdom teeth out. And I'm diabetic so I have periodontia. And they won't do anything, they won't do any coverage ‘til you're in it for a complete year. Now that I have that lapse now I'm not covered for getting my wisdom teeth out for another year. They are sitting in my face rotting… I have like an 80 dollar previous bill so they won't even make me an appointment.” [508]. Previously made dental appointments were skipped, and interviewees reported not being likely to seek dental care for future issues.

Financial hardships caused by disenrollment were a major theme in the interviews. Even with high levels of employment, many interviewees stated that being disenrolled created a burden and barriers to receiving medical or dental care, obtaining prescriptions, or obtaining new insurance. Interviewees reported lacking health insurance even for a short time caused worry about emergency care and not being able to obtain needed medication or seek routine care. One interviewee related this saying, “Well, it's kinda scary, not havin’ insurance but I haven't been without it for very long at a time, but you know, I couldn't get my medicine or anything like that.” [732]. Interviewees' reactions to disenrollment ranged from mild frustration with little negative impact to severe stress with significant negative impact.

When the survey respondents were asked if they did anything to prepare for disenrollment, almost 90% did nothing (Table 4). Survey respondents engaged in behaviors that could be detrimental including delaying filling prescriptions, skipping medication doses, stopping taking medications, and not seeking health care or dental care (Table 3).

#### On their subsequent health insurance status

No interviewees were able to appeal their disenrollment, instead often opting to re-enroll. One interviewee expressed feeling insecure about re-enrollment: “They got me back on for now, but I don't know how long that's gonna last.” [846]. Interviewees who were able to re-enroll in IHAWP or obtain other health insurance experienced fewer challenges, less stress and/or anxiety, and less confusion compared with those who were unable to obtain coverage. Interviewees had mixed success at getting re-enrolled in IHAWP or finding other insurance. Twenty-nine had successfully re-enrolled, and 12 obtained alternate insurance. Although some interviewees stated that re-enrolling was easy and the process was clear, others noted difficulties or concerns, such as having to try multiple times to re-enroll, being uninsured while the re-enrollment process took time, being denied coverage when they re-enrolled, or having to take time off work to re-enroll. One interviewee voiced the frustration with accessing assistance to understand the process: “I call customer service DHS [Department of Health Services], then they send me to a different person, then they send me to a different person. They just need one person, like, is in charge of my case. Instead of going around the merry-go-round. That's just crazy. And nobody has the answers.” [633].

Asked if they had been uninsured for a period of time, 81.3% of survey respondents said yes. Over two-fifths of the respondents stated they had no insurance (Table 2). After they were disenrolled, some took action, including calling to re-enroll (19.6%), going online to re-enroll (12.9%), and going in person to re-enroll (7.1%), but about two-fifths of the respondents did nothing after being disenrolled (Table 4). Very few (3.1%) respondents appealed their disenrollment, and 13.3% looked for new insurance. Respondents who did something to prepare for being disenrolled and who did something after being disenrolled were more likely to be re-enrolled in any health insurance program compared with those who did nothing (Table 4). We asked those who had been able to re-enroll about the ease of the process, and 38.1% stated it was difficult or very difficult.

## Discussion

Our mixed methods study involved in-depth interviews and a mailed survey with individuals disenrolled from IHAWP, Iowa's waiver-based Medicaid expansion. We examined the factors that contributed to individuals' disenrollment, their experience of being disenrolled, and the impact of disenrollment on their health and health insurance status. This study is one of the few that documents disenrollment, using both qualitative and quantitative methods for a rich description of how people become disenrolled, what they try to do about it, and the consequences disenrollment has on them. These findings have concrete implications for states currently implementing or considering a disenrollment component to their 1115 Waiver.

Our results showed the significant, negative consequences that disenrollment can have on Medicaid enrollees. Nearly two-thirds (64.6%) of disenrolled individuals were not re-enrolled in any health insurance program and lacking health insurance negatively impacted them. This is especially important to consider given the number of respondents reporting multiple chronic health conditions. The respondents noted significant stress and anxiety related to their disenrollment and a reduced ability to manage their health. Those who were unable to obtain health insurance coverage took potentially harmful actions, such as stretching medication and delaying or forgoing needed medical or dental care. For those that might have been about to enrollee in some other health insurance, it is likely the health insurance would not provide dental coverage, leaving people vulnerable. These results were similar to other studies showing negative impacts on disenrolled individuals' finances,^9^ re-enrollment in health insurance,^10,11^ and health care utilization and access.^6,8,13^ As states consider disenrollment options, understanding the consequences to disenrolled members is vital to ensure the program does not create further disparities.

Our findings underscore the importance of effectual implementation of personal responsibility requirements, especially if it can lead to disenrollment, and are important for states planning implementation of their programs. Numerous interviewees and respondents in this study were unaware of the HBP, did not know they owed a premium, and were unaware they could claim financial hardship to avoid the required premium. Furthermore, many interviewees and respondents were not able to correctly identify why they were disenrolled. These findings are in line with previous research on the HBP that found a high level of confusion among enrollees regarding HBP requirements.^3^ Given that ∼45% of survey respondents said they failed to pay the monthly premium because they did not have the money, finding ways to increase HBP participation or ensure enrollees are aware of the financial hardship exemption could prevent both disenrollment and the consequent stress on IHAWP enrollees.

We can look to strategies used to work with traditionally hard to reach or vulnerable populations to offer insight into how states can best convey complicated information about health insurance. Patient navigation has been used to improve health care delivery to disadvantaged groups by offering support needed to successfully utilize the health care system.^14,15^ The patient navigator ensures that barriers do not prevent people from receiving the care they need.^16^ Patient navigation has been used with Medicaid populations to increase completion of preventive health behaviors,^17–19^ which is the ultimate goal of the HBP. States should consider the lessons learned from this model to better serve Medicaid members, allow them to successfully navigate the health care system, and prevent negative outcomes.

States should also consider the incentive mechanism of personal responsibility programs, and whether to use rewards or penalties to motivate members.^20^ The HBP frames itself as a reward for completing healthy activities, but instead imposes a penalty, a premium, to members who do not engage in those activities. Furthermore, it has an additional penalty, disenrollment, for individuals who do not pay the premium. Rewards and penalties need to be designed based on an understanding of why people do or do not engage in the desired behavior and what are the trade-offs for changing their behaviors.^20^ Moreover, penalties are more appropriate for dissuading individuals from doing something, rather than encouraging a new behavior.^20^ Taken in context of the results presented here, perhaps using carrots rather than sticks would avoid many of the negative outcomes associated with disenrollment, reduce the administrative burden and costs associated with disenrollment, and more effectively encourage completion of the program activities.

Our study had some limitations. First, nonresponse bias may affect our results, because interviews and surveys can only capture the experiences and perceptions of those who participate. There may be selection bias as individuals who choose to respond may be motivated by particularly good or bad experiences that make them less representative of the typical individual. Finally, because we rely on self-reported data, the information we collect is potentially subject to recall bias and social acceptability bias. Although it is difficult to assess the extent to which such bias may be present, the results of the interviews and survey data aligned well with each other and previous research findings, increasing our confidence in the results.

## Conclusion

Disenrollment had significant, negative impacts on individuals, including financial difficulties, stress and anxiety, adverse health impacts, and inability to obtain health insurance coverage. These impacts could be mitigated by better informing enrollees of both the program requirements and their option to claim financial hardship when owing a premium. If there are limited resources for communicating with enrollees, states may want to reconsider the program.

Understanding the circumstances that lead to disenrollment can inform strategies for preventing disenrollment. Further research on communicating with hard-to-reach populations about these complex issues is required. Future researchers can provide valuable evidence to decision makers by evaluating IHAWP's efforts to prevent disenrollment or alleviate its negative effects, and examining the long-term impact of disenrollment on individuals' health and health insurance coverage. Long-term future research could also examine how disenrollment from Medicaid affects the finances of health systems and hospitals because, as the health of disenrolled individuals deteriorates, there may be more unpaid care provided by health systems and hospitals.

## Abbreviations Used

FPL: federal poverty level
HBP: Healthy Behaviors Program
IHAWP: Iowa Health and Wellness Plan
